# The impact of colon injuries on the outcome of gunshot wounds to the abdomen

**DOI:** 10.1007/s00423-023-03067-0

**Published:** 2023-08-23

**Authors:** Yonita Singh, Sooraj Motilall, Bongani L. Khulu, Brandon S. Jackson

**Affiliations:** 1 Department of Surgery, Tembisa Provincial Tertiary Hospital, University of Pretoria, Pretoria, 0007 South Africa; 2 Department of Surgery, Steve Biko Academic Hospital, University of Pretoria, Pretoria, 0007 South Africa; 3 Department of Surgery, Kalafong Provincial Tertiary Hospital, University of Pretoria, Pretoria, 0007 South Africa

**Keywords:** Gunshot injuries, Colon-related complications, Morbidity, Mortality

## Abstract

**Purpose:**

Factors predicting morbidity and mortality in patients with colon-related gunshot injuries and the management of these injuries are not always straightforward. This aimed to assess the impact of abdominal gunshot wounds with colonic injuries on patients’ overall outcomes.

**Methods:**

This cross-sectional prospective observational study compared patients with colon injuries and without colon injuries. Data was collected from admission, theatre and postoperative care. Patients were recruited between 1 January 2020 and 20 October 2021.

**Results:**

Of 132 patients with abdominal gunshot injuries, 71 (54.0%) had colon injuries. The colon injury group had a higher incidence of laparotomy wound sepsis (*p*<0.0001), bullet exit wound infection (*p*<0.0001), tract necrotizing fasciitis (*p*<0.0001), relook laparotomies (*p*<0.0001) and a longer hospital stay (*p*<0.0001). Septicaemia (*p*=0.002) or anastomotic leak (*p*=0.041) was associated with a penetrating abdominal trauma index (PATI) ≥25. Most patients who developed tract necrotizing fasciitis did not have their tract debrided/ lavaged (*p*=0.004). The type of colon repair did not influence the length of hospital stay (*p*=0.688) or the development of a colon-related complication (*p*=0.578). Between 18 and 25 years (*p*<0.0001) and >2 organs injured (*p*=0.018) were associated with colon-related complications. Patients between 18 and 25 years were 4.748 times more likely to develop a colon-related complication (*p*=0.046).

**Conclusion:**

Gunshot wounds to the abdomen with associated colonic injuries had a worse outcome with an increased risk of developing wound infections. There is no difference in the operative management of colonic injury. Patients between the ages of 18 and 25 years or >2 organs injured are more likely to develop a complication.

## Introduction

Gunshot injuries pose an immense danger due to the high kinetic energy of bullets which causes damage locally and in the surrounding tissues. Since the initial trajectory of the projectile may change after entering the body, damage to distant organs can occur [1]. Abdominal injuries from gunshot wounds occur in 49.0 to 64.0% [2]. Small bowel appears to be the abdominal organ most frequently injured, followed by the colon and liver [3]. The morbidity from gunshot-associated colon injuries has been reported to be as high as 47.0% [3–6]. The majority of these patients will have faecal contamination of the peritoneal cavity [4]. Colon injury-related mortality has been reported up to 21.7%. Potential contributing risk factors include shock on admission, faecal contamination, duration of operation >4 h, more than two postoperative complications, and a penetrating abdominal trauma index (PATI) score >25. [4] A high PATI score is associated with a seven-time increase in risk of mortality [6]. A PATI score >15 has been reported to be associated with twentyfold increased mortality rate [3], whereas others have reported >25 as a better predictor of mortality in penetrating abdominal trauma [6, 7]. The rate of postoperative complications increases sharply if the PATI is >25 [8].

There are multiple controversial issues regarding the management of colon injuries. These include time to definitive care, management of the gunshot tract, and management of the colonic injury, i.e. primary repair vs. colostomy. Management algorithms for colonic trauma have evolved since the Second World War [9]. Penetrating civilian colon injuries are less destructive than their military counterparts. The trend has changed to primary colon repair instead of mandatory diversion, specifically for non-destructive colon injuries [9, 10]. There does not appear to be a significant difference in outcome between patients who had primary repair and those undergoing diverting colostomies [4, 11]. The emergence of damage control surgery towards the end of the twentieth century once again influenced management. The principle of damage control surgery is to avoid all definitive surgery in the unstable patient at the initial operation [12]. Some reports show that primary diversion is still the preferred method over primary anastomosis [13].

The management of the bullet tract is also controversial. Sepsis was five times greater in those with retained bullets and seven times greater where the bullet tract was not debrided. The incidence of sepsis appears to decrease when a retained bullet, that had penetrated the colon, was surgically removed [14]. The bullet should therefore be removed if feasible and the local tissue debrided after an abdominal gunshot wound with colon injury. It has been advocated that if the bullet has exited spontaneously, the tract should be debrided and lavaged extensively [14].

Factors predicting morbidity and mortality in patients with gunshot injuries to the colon and the management of those injuries are not always straightforward. Investigating as many factors as possible may possibly assist to prognosticate or even identify high-risk patients earlier and possibly improve management to prevent colon-related morbidity. Therefore, the aim of the study was to assess the impact of abdominal gunshot wounds with colonic injuries on the patient’s overall outcome. The objectives were to determine the prevalence of colon-related complications and to determine what risk factors contribute to increased morbidity and mortality.

## Methods

The study was performed as a cross-sectional prospective observational study. Patients were recruited from three tertiary academic hospitals affiliated with the University of Pretoria, i.e. Steve Biko Academic Hospital, Tembisa Provincial Tertiary Hospital, and Kalafong Provincial Tertiary Hospital. All patients 18 years and older with gunshot injuries to the abdomen that underwent a surgical exploration, where a breach of the peritoneal cavity was found, were included. Patients were excluded if they were operated on at another institution and thereafter presented with post-operative complications. Data was collected between 1 January 2020 and 20 October 2021.

Patients with colon injuries were compared to those with other hollow-viscus organ injuries, excluding colonic. Admission parameters were recorded and included respiratory rate, heart rate, blood pressure, shock index (heart rate/systolic blood pressure), Glasgow coma scale, and location of entry/exit wounds. Blood gas parameters were categorised based on indications for damage control surgery, that are associated with a poorer prognosis, such as pH<7.2, lactate >5 and serum base excess <-5.

Operative data included operating time, hollow viscus organ injury, presence of faecal contamination, PATI score, blood product transfusion in the first 24–48 h, gunshot wound tract management, damage control vs. definitive surgery, colon injury surgical management (i.e. primary repair/resection and anastomosis or colon diversion) and the number of relook laparotomies. Faecal contamination, site, and number of penetrating colon injuries were also recorded. Faecal contamination was defined as minimal if there was spillage confined to the immediate area around the injury, moderate when spillage was confined to one quadrant of the abdomen, and major if faecal contamination was found in more than one quadrant [4]. Post-operative complications and follow-up, until death or discharge or up to 60 days post-discharge, were also recorded.

Informed consent was obtained from patients on admission by the admitting/ operating doctor, provided the patient had a Glasgow coma scale of 15/15, or at any time during their hospital stay once they were stable. If the patient was unable to consent then the next of kin or the custodian of data, i.e. the Chief Executive Officer (CEO) of the respective hospital, was requested for informed consent. Consent was also obtained from all the relevant hospitals in order to perform the research. The research was registered with the Nation Health Research Database (NHRD), South Africa (registration number GP_202002_002). This study was performed in line with the principles of the Declaration of Helsinki. Ethics approval was obtained from the University of Pretoria, Faculty of Health Sciences, Research Ethics Committee (reference no: 789/2019).

### Statistical analysis

Descriptive statistics were determined for the data, including mean and standard deviation for continuous data and proportions for categorical data. Frequencies were represented as percentages. The chi-square test was used to assess the association between categorical variables. Logistic regression was conducted as a multivariate technique to establish the variables that were significant in predicting colon-related complications. The variables that were found to be significant with univariate statistical analysis, were used as independent variables in the logistic regression model. All statistics were evaluated at a 5% level.

## Results

A total of 132 patients with abdominal gunshot injuries were recruited. The majority, 128 (96.97%), were males with only 4 (3.03%) females. The mean age was 33 years. Seventy-one (54.0%) had associated colon injuries.

Complications in patients with injuries to the colon were compared to those with other hollow-viscus organ injuries but excluding colon, i.e. non-colon injury (Table 1). The colon injury group had an overall higher incidence of complications compared to the non-colon injury group. The complications that were significantly higher in the colon injury group included laparotomy wound sepsis (*p*<0.0001), bullet exit wound infection (*p*<0.0001), and the development of bullet tract necrotizing fasciitis (*p*<0.0001). The rest of the complications, although had a greater trend in the colon injury group, were not statistically significant between the two groups.

**Table 1:** Complications associated with colon injuries

Complication		Colon injury		Total *n* (%)	*p*-value
		No *n* (%)	Yes *n* (%)		
Laparotomy wound sepsis	Yes	14 (23)	36 (50.7)	50 (37.9)	<0.0001
	No	47 (77)	35 (49.3)	82 (62.1)	
Septicaemia	Yes	3 (4.9)	7 (9.9)	10 (7.6)	0.339
	No	58 (95.1)	64 (90.1)	122 (92.4)	
Anastomotic leak	Yes	1 (1.6)	6 (8.5)	7 (5.3)	0.123
	No	60 (98.4)	65 (91.5)	125 (94.7)	
Intra-abdominal abscess	Yes	4 (6.6)	13 (18.3)	17 (12.9)	0.066
	No	57 (93.4)	58 (81.7)	115 (87.1)	
Bullet exit wound infection	Yes	3 (4.9)	24 (33.8)	27 (20.5)	<0.0001
	No	58 (95.1)	47 (66.2)	105 (79.5)	
Tract necrotising fasciitis	Yes	1 (1.6)	15 (21.1)	16 (12.1)	<0.0001
	No	60 (98.4)	56 (78.9)	116 (87.9)	
Nosocomial infection	Yes	7 (11.5)	9 (12.7)	16 (12.1)	1.000
	No	54 (88.5)	62 (87.3)	116 (87.9)	
60-day follow-up	Refused treatment	2 (3.3)	1 (1.4)	3 (2.3)	0.595
	Death	12 (19.7)	15 (21.1)	27 (20.5)	1.000
	Discharge	13 (21.3)	6 (8.5)	19 (14.4)	0.047
	60 days	34 (55.7)	49 (69)	83 (62.9)	0.149

Forty-seven patients required a relook laparotomy of which 23 (48.9%) were as-required/on-demand and 24 (51.1%) were planned. The colon injury group had significantly more patients who required relook laparotomies, 33 (70.21%) compared to 14 (29.79%) (*p<*0.0001). Of the patients with colon injuries requiring relook laparotomy, 24 (72.7%) were due to colon-related complications that required reintervention. Some patients in the colon injury group required more than just one relook laparotomy mainly due to ongoing sepsis. Sixteen patients required one-, 13 patients required two-, three patients required four- and one patient required six- relook laparotomies. The three main indications for the relook laparotomies in the colon injury group were to address an anastomotic leak (and complications thereof), washout of intra-abdominal collections and debridement of the gunshot tract necrotizing fasciitis or laparotomy wound sepsis. Patients with colon injuries had a significantly longer hospital stay of 17 days compared to 8 days in the non-colon injury group (*p*<0.0001). Complications compared to the PATI score (Table 2) after gunshot abdominal injuries demonstrate that septicaemia (*p*=0.002) or an anastomotic leak (*p*=0.041) were more likely if the PATI score was ≥25.

**Table 2:** Abdominal gunshot complications compared to the Penetrating abdominal trauma index (PATI) score

Variable	Colon injury	PATI			p-value
		<25 (*n* =88) *n* (%)	≥25 (*n* =44) *n* (%)	Total (*n* =132) *n* (%)	
Laparotomy wound sepsis	Yes	32 (36.4)	18 (40.9)	50 (37.9)	0.704
	No	56 (63.6)	26 (59.1)	82 (62.1)	
Septicaemia	Yes	2 (2.3)	8 (18.2)	10 (7.6)	0.002
	No	86 (97.7)	36 (81.8)	122 (92.4)	
Anastomotic leak	Yes	2 (2.3)	5 (11.4)	7 (5.3)	0.041
	No	86 (97.7)	39 (88.6)	125 (94.7)	
Intra-abdominal abscess	Yes	8 (9.1)	9 (20.5)	17 (12.9)	0.096
	No	80 (90.9)	35 (79.5)	115 (87.1)	
Bullet exit wound infection	Yes	17 (19.3)	10 (22.7)	27 (20.5)	0.653
	No	71 (80.7)	34 (77.3)	105 (79.5)	
Tract necrotising fasciitis	Yes	8 (9.1)	8 (18.2)	16 (12.1)	0.160
	No	80 (90.9)	36 (81.8)	116 (87.9)	

The final outcome of the two groups of patients in terms of refused hospital treatment, mortality, recovered, discharged and followed-up at 60 days (either at the surgical clinic or telephonically) were analysed. Of the 132 patients, 3 patients signed a refusal of hospital treatment post-surgery (2.3%) and 27 (20.5%) died. The mortality rate between the colon and non-colon injury groups (21.1% vs. 19.7%) were fairly similar. In terms of follow-up, 19 patients (14.4%) were discharged but did not return for their follow-up appointments and were also not contactable telephonically. However, 83 patients (62.9%) were followed up at the surgical clinic at 60 days or were contacted telephonically.

### Colon-related complications

From the 71 patients who had colonic injuries as seen in Table 1, 52 (73.2%) had at least one colon-related complication and 19 (26.8%) had none. Some patients developed multiple colon-related complications. The most common complications encountered were laparotomy wound sepsis, which occurred in 36 patients (50.7%) followed by bullet exit wound infection in 24 patients (33.8%), tract necrotizing fasciitis in 15 patients (21.1%) and intra-abdominal abscess formation in 13 patients (18.3%).

In order to determine potential risk factors that predict the development of colon-related complications, multiple variables were analysed. The majority of the patients were male (95.8%) and their age distribution was fairly even between the 3 ranges: 18-25, >25–<35, and ≥35. Forty-two patients (59.2%) arrived less than 2 h after sustaining the actual gunshot injury whereas the remaining 29 (40.8%) arrived more than 2 h after the injury. At the definitive operating hospital, delays were minimised as far as possible and 49 patients (69.0%) were taken to the operating room and operated on within 1–4 h of arrival. Of the 6 patients who had an in-hospital theatre delay >9 h, 2 were due to the patients not consenting for surgery and the remaining 4 were due to busy month-end weekends that had an overwhelming number of emergency cases awaiting theatre.

The data from vital signs and blood gas parameters were on arrival to the emergency department. Shock index was calculated by looking at the patient’s first blood pressure and pulse measurements on arrival at the hospital (Shock Index= HR/ SBP). Forty-five patients (63.1%) of the colon injury group of patients were within the mild shock category. Blood gas parameters that were analysed included pH, lactate, serum base excess, and bicarbonate (HCO3) levels. Cut-off values used for this study were based on guidelines for damage control surgery indications and values on arrival that are usually associated with poorer prognosis in patients who sustain penetrating abdominal injuries. These included values such as pH<7.2, lactate >5 and serum base excess <−5/−6. Only 1 patient did not have a blood gas result on arrival at the emergency department.

Some patients sustained injuries to multiple sites of the colon. The most common area on the colon that was injured was the transverse colon in 35.2% of patients. Only 10 patients (14.1%) had a total operating time of less than 90 min. Amongst these 10, were patients that either had a simple colon injury requiring a debridement and primary repair, or patients that underwent damage control surgery in the form of a resection and “clip and drop” of that segment of the colon.

The degree of faecal contamination was documented in all patients who sustained a colonic injury. This was graded into no contamination, minimal, moderate, or major contamination. The majority of patients had no contamination or minimal contamination. Both categories combined comprised over 70.0% of the total group.

The majority of patients with colon injuries did not have blood transfusion (64.8%) or the use of only 2 units of red-packed cells (21.1%). With regards to bullet tract management, 84.5% of patients did not have their gunshot tract debrided/lavaged on the index laparotomy. With regards to bullet exit wound management, 69.0% did not have their exit wounds debrided.

The repair method of the colon injury was assessed. There was a fairly similar distribution of the repair methods used. A large proportion (43.7%) of the injuries were simple colon injuries that were debrided and primarily repaired. Anastomotic leak was present in 6 (8.5%) patients (see Table 1) and this required these patients to have a relook laparotomy with resection of that leaking anastomosis and creation of a stoma.

Data was analysed in the colon injury group with regard to potential risk factors that could predict a colon-related complication (Table 3). The PATI score of ≥25 was not associated with the risk of developing a colon-related complication (*p*=0.793). The only two risk factors that were significantly associated with developing a colon-related complication were having ≥2 organs injured (*p*=0.018) and the age category 18–25 years (*p*<0.0001). From the logistic regression model, patients with colon injuries aged 18–25 years were 4.748 times more likely to have a colon-related complication compared to those that were >35 years old (*p*=0.046). The number of organs injured was not significant in predicting complications when using multivariate statistics.

**Table 3:** Univariate analysis of potential predictors of morbidity in patients exposed to injuries of the colon

Variable	Option	Colon-related complication			*p*-value
		None (*n*=19) *n* (%)	At least 1 (*n* =52) *n* (%)	Total (*n* =71) *n* (%)	
Sex	Male	19 (100)	49 (94.2)	68 (95.8)	0.559
	Female	0 (0)	3 (5.8)	3 (4.2)	
Age category	18–25	3 (15.8)	19 (36.5)	22 (31)	<0.0001
	26–34	6 (31.6)	22 (42.3)	28 (39.4)	
	≥35	10 (52.6)	11 (21.2)	21 (29.6)	
Smoking	Yes	8 (50)	26 (55.3)	34 (54)	0.777
	No	8 (50)	21 (44.7)	29 (46)	
Injury to arrival time	>2 hours	6 (31.6)	23 (44.2)	29 (40.8)	0.422
	1–2 hours	9 (47.4)	16 (30.8)	25 (35.2)	
	<1 hour	4 (21.1)	13 (25)	17 (23.9)	
Arrival time to surgery	≥9 hours	3 (15.8)	3 (5.8)	6 (8.5)	0.104
	5–8 hours	0 (0)	11 (21.2)	11 (15.5)	
	1–4 hours	15 (78.9)	34 (65.4)	49 (69)	
	>1 hour	1 (5.3)	4 (7.7)	5 (7)	
Shock index on arrival	≥1.4 (Severe Shock)	0 (0)	2 (3.8)	2 (2.8)	0.188
	1–<1.4 (moderate Shock)	0 (0)	9 (17.3)	9 (12.7)	
	0.6 – <1 (mild shock)	14 (73.7)	31 (59.6)	45 (63.4)	
	<0.6 (no shock)	5 (26.3)	10 (19.2)	15 (21.1)	
pH ≤7.2	Yes	2 (10.5)	3 (5.9)	5 (7.1)	0.608
	No	17 (89.5)	48 (94.1)	65 (92.9)	
Serum base excess ≤-5	Yes	8 (42.1)	23 (45.1)	31 (44.3)	1.000
	No	11 (57.9)	28 (54.9)	39 (55.7)	
Lactate	>5	3 (15.8)	14 (27.5)	17 (24.3)	0.126
	2–5	15 (78.9)	27 (52.9)	42 (60)	
	<2	1 (5.3)	10 (19.6)	11 (15.7)	
HCO3 ≤22	Yes	12 (63.2)	33 (64.7)	45 (64.3)	1.000
	No	7 (36.8)	18 (35.3)	25 (35.7)	
Number of organs injured	1	4 (21.1)	11 (21.2)	15 (21.1)	0.018
	2	3 (15.8)	23 (44.2)	26 (36.6)	
	3	7 (36.8)	14 (26.9)	21 (29.6)	
	4	2 (10.5)	4 (7.7)	6 (8.5)	
	5	3 (15.8)	0 (0)	3 (4.2)	
Colonic injury site	Caecum	1 (5.3)	9 (17.3)	10 (14.1)	0.270
	Ascending colon	0 (0)	3 (5.8)	3 (4.2)	0.559
	Hepatic flexure	2 (10.5)	4 (7.7)	6 (8.5)	0.655
	Transverse	9 (47.4)	16 (30.8)	25 (35.2)	0.263
	Splenic flexure	2 (10.5)	3 (5.8)	5 (7)	0.605
	Descending	2 (10.5)	4 (7.7)	6 (8.5)	0.655
	Sigmoid	4 (21.1)	10 (19.2)	14 (19.7)	1.000
	Rectum	1 (5.3)	12 (23.1)	13 (18.3)	0.162
Total operating time ≤90 minutes	No	17 (89.5)	44 (84.6)	61 (85.9)	0.719
	Yes	2 (10.5)	8 (15.4)	10 (14.1)	
Degree of faecal contamination	Major	3 (27.3)	6 (17.1)	9 (19.6)	0.729
	Moderate	3 (27.3)	8 (22.9)	11 (23.9)	
	Minimal	5 (45.5)	21 (60)	26 (56.5)	
	No Contamination	8 (42.1)	17 (32.7)	25 (35.2)	
Units of red packed cells used intra-operatively	> 4	1 (5.3)	0 (0)	1 (1.4)	0.268
	≤ 4	18 (94.7)	52 (100)	70 (98.6)	
Gunshot wound tract lavaged/ debrided	No	17 (89.5)	43 (82.7)	60 (84.5)	0.715
	Yes	2 (10.5)	9 (17.3)	11 (15.5)	
Entry and exit wounds debrided	No	14 (73.7)	35 (67.3)	49 (69)	0.774
	Yes	5 (26.3)	17 (32.7)	22 (31)	
Penetrating abdominal trauma index (PATI)	≥25	10 (52.6)	25 (48.1)	35 (49.3)	0.793
	<25	9 (47.4)	27 (51.9)	36 (50.7)	
Resection and “clip & drop” (Damage control surgery)	Yes	4 (21.1)	8 (15.4)	12 (16.9)	0.722
Debrided and Primary Repair	Yes	8 (42.1)	23 (44.2)	31 (43.7)	1.000
Resection and Anastomosis	Yes	5 (26.3)	12 (23.1)	17 (23.9)	0.762
Resection and Stoma	Yes	5 (26.3)	18 (34.6)	23 (32.4)	0.578

## Discussion

Colon injuries from abdominal gunshot injuries can occur in up to 40–50% of patients [3], which is reflected in our study where 71 patients (54.0%) sustained colon injuries. The majority of the colon-related complications were infection-related, most commonly due to the predominance of faecal contamination with gram-negative organisms [15]. The incidence of laparotomy wound sepsis (*p*<0.0001) and bullet exit wound infection (p<0.0001) was significant in the colon injury group. The concerning complication was the development of bullet tract necrotizing fasciitis in 21.0% of patients in the colon injury group compared to only 2.0% in the non-colon injury group (*p*<0.0001). This can be explained by the phenomenon that as a bullet travels through soft tissue, a vacuum is created as permanent and temporary cavities are formed [16]. This vacuum suctions potential infective sources from the exterior. Similarly, as the bullet penetrates an unprepared colon, gained bacteria are deposited throughout the exit pathway of the projectile. This highlights the importance of gunshot wounds and tract management. The bullet should be removed, if feasible, and the gunshot wound and bullet tract should be debrided and extensively lavaged [14]. In the group of patients with colon injuries, 84.5% of patients did not have their gunshot tract debrided/lavaged on the initial laparotomy and 69.0% did not have their entry and exit wounds debrided. Necrotizing fasciitis of the bullet tract occurred in 20 patients (95.2%) who did not have their tract debrided/ lavaged (*p*=0.004). Debridement and lavage of gunshot wounds and tracts should therefore be standard practice at every index laparotomy for abdominal gunshot injuries.

The mean length of hospital stay for patients with associated colon injury was significantly longer by almost 9 days (*p*<0.0001). This can be attributed to the higher incidence of complications, with some patients requiring multiple reinterventions and/or specialized wound care. Morbidity from colonic injury has been associated with extended hospitalization, increased health care costs, prolonged antibiotic coverage, prolonged critical care stay and higher mortality [5]. Of the 33 patients who had relook laparotomies in the colon injury group, 72.7% were directly due to colon-related complications. Almost half required ≥2 subsequent laparotomies mainly for anastomotic leak (and complications thereof), washout of intra-abdominal collections and multiple debridements of tract necrotizing fasciitis. It was noted that debridement for tract necrotizing fasciitis was occasionally not adequate after one debridement which resulted in multiple operations.

The in-patient mortality rate for patients with abdominal gunshot injuries was 20.5% (27 patients out of 132), which was about 5% higher than previous reports [17]. The mortality was fairly similar between the colon and non-colon injury groups (21.1% vs 19.7%). This highlights the concept from Saar et al. that colon injury-associated mortality is related to overall injury burden and haemorrhage rather than to the actual colon injury itself [18].

The majority of patients (73.2%) with associated colon injuries had at least one colon-related complication, with more than half having 2 or more. The incidence of colon-related complications was higher than previously reported at 38.9% [19]. The most common complications encountered were laparotomy wound sepsis (50.7%) followed by bullet exit wound infection (33.8%), tract necrotizing fasciitis (21.1%), and intra-abdominal abscess formation (18.3%). The high rate of complications can be attributed to the fact that the preventative measures mentioned, i.e. debridement/lavage of bullet exit wounds and bullet tracts, were not practiced routinely.

A PATI score ≥25 is associated with a higher rate of postoperative complications [8]. Similar results were seen in our overall study population, where PATI ≥25 was associated with a higher incidence of complications, specifically with septicaemia (*p*=0.002) or an anastomotic leak (*p*=0.041). However, when specifically observing the 71 patients with associated colon injuries, PATI ≥25 was not statistically significant as a predicting risk factor for colon-related complications (*p*=0.793). This may be due to a small sample size. The PATI score was also not specifically designed to predict colon-related complications directly but rather for postoperative complications in general.

Besides the PATI score, other certain factors are considered as predictors for colon-related complications and mortality including multiple blood transfusions, >2 organs injured, shock on admission, degree of faecal contamination and duration of the operation >4 h [4, 8, 17, 19]. Blood product transfusion ≥4 units within 24 h has been associated with increased colon-related complications [8]. However, no conclusion could be drawn from the impact of transfusion in our study as the majority (98.6%) of patients with colon injuries had ≤4 units of transfusion. Only two risk factors had a significant association with developing colon-related complications, i.e. two or more organs injured (*p*=0.018) and the age category 18–25 years (*p*=0.0001). The degree of associated injury appears to be more important to the development of infectious complications rather than the type of colon repair performed. From the logistic regression model, patients with colonic injuries aged 18–25 years were 4.748 times more likely to have at least one colon-related complication. Contributing factors may have negatively impacted the patients in this age group which increased the likelihood of developing colon-related complications. High-risk factors included the majority were smokers, more than one site of their colon was injured, the majority did not have their trauma wounds or tracts lavaged/debrided, injury to arrival time was >2 h, the presence of a high degree of faecal contamination and patients presented with more extensive injuries resulting in, at least, mild to moderate shock.

The surgical method used to repair colonic injuries may influence colon-related complications. As civilian-related gunshot wounds are caused by low-velocity handguns, it is reasonable to perform primary colonic repair or resection with anastomosis [10]. There was a fairly similar distribution of the repair methods, i.e. primary repair/anastomosis or diversion, used in the colonic injury group with 43.7% having simple colon injuries that were debrided and primarily repaired. The anastomotic leak occurred in 8.5% of patients resulting in a relook laparotomy with resection of the leaking anastomosis and a stoma formation. No repair method was significant in predicting the development of colon-related complications (*p*=0.578) or influencing the length of hospital stay (*p=*0.688).

When analysing delays from injury to theatre, although 59.0% of patients arrived less than 2 h after sustaining the gunshot injury, 40.8% arrived with more than 2 h delay. The delays reflect multiple prehospital factors such as an inadequate number of emergency service vehicles. The initial receiving hospital may not be the most appropriate facility with the required surgical expertise and thereby necessitating further transportation to definitive care. Also, peripheral clinics and district hospitals are staffed predominantly by junior doctors who may not appreciate the severity of the injury and the need for urgent surgical intervention. Unfortunately, achieving definitive care within the golden hour is challenging, especially in third-world countries. [20] Although the majority of patients were taken to theatre within 1–4 h, some patients had delays of >9 h. The reasons for the delays were due to the patients not consenting to surgery, the high volume of patients requiring emergency surgery, and prioritising emergency conditions for theatre time amongst the various surgical disciplines. This highlights the impact of the burden of trauma on the healthcare system. Theatre delays results in increased mortality and a higher incidence of infectious complications [3]. Surprisingly, delays in definitive management failed to influence colon-related complications or mortality in our study. This may be explained by patients who survive the journey to the hospital, or in-hospital delays, had injuries that were probably less severe, whereas those with other fatal injuries may have died from their injuries prior to their arrival at the hospital.

Strengths of this study include a prospective design to address the aims and objectives. As the study was observational, the surgeon’s management was not influenced and therefore reflects routine clinical practice. Unfortunately, the majority of the data was collected during the peak of the COVID-19 pandemic. Therefore, the limitations of the study included the number of patients recruited over this period was not a true reflection of the actual number that would normally be encountered. The data was also limited to the patients admitted to the three tertiary academic hospitals. The extent of faecal contamination, although defined, was subjective. Risk factors which could affect the results, such as comorbidities, immunosuppressant use, drug abuse, etc., were not investigated.

## Conclusion

Gunshot wounds to the abdomen with associated colonic injuries had a worse outcome with an increased risk of developing wound infections. There is no difference in the operative management of colonic injury. Patients between the ages of 18–25 years or >2 organs injured are more likely to develop a complication.

## Data Availability

The dataset(s) supporting the conclusions of this article are available from the authors.
